# Performance of renin assays in selecting fludrocortisone dose in children with adrenal disorders

**DOI:** 10.1530/EC-23-0370

**Published:** 2024-01-22

**Authors:** Timothy J Morris, Andrew Whatmore, Laura Hamilton, Beverly Hird, Eric S Kilpatrick, Lesley Tetlow, Peter Clayton

**Affiliations:** 1 Directorate of Biochemistry, Manchester University NHS Foundation Trust, Manchester, UK; 2 Faculty of Biology Medicine and Health, University of Manchester, Manchester, UK; 3 Division of Developmental Biology and Medicine, University of Manchester, Royal Manchester Children’s Hospital, Manchester, UK; 4 Pathology Department, Clinical Biochemistry, Huddersfield Royal Infirmary, Lindley, Huddersfield, UK

**Keywords:** renin assays, fludrocortisone, children, adrenal disorders

## Abstract

Children with salt-wasting adrenal insufficiency are managed with glucocorticoid and mineralocorticoid replacement. Measurement of renin activity or concentration alongside blood electrolyte levels is used to monitor the adequacy of mineralocorticoid replacement. Our unit changed from using renin activity to renin concentration and carried out a service review to assess whether this influenced decision-making for fludrocortisone dosing. In total, 50 measurements of plasma renin activity and 50 of renin concentration were analysed on separate cohorts before and after the assay change, with values standardised to multiples of the upper limit of normal (MoU) to allow comparison between assays. We were more likely to increase the fludrocortisone dose for a raised renin concentration than a raised renin activity. The renin concentration MoU was more strongly related to plasma sodium (negatively) and 17α-hydroxyprogesterone (17α-OHP) (positively) than the renin activity MoU. Using a MoU cut-off of 1.5, a decision to increase the dose of fludrocortisone was more likely to be made when using the renin concentration assay compared with the activity assay. Using a cut-off of 40 nmol/L for 17α-OHP, a decision not to change the fludrocortisone dose when 17α-OHP was <40 was more likely when using the renin concentration assay. For both assays, a plasma sodium <140 mmol/L was more likely to lead to a fludrocortisone dose increase, and most likely for the renin concentration assay. Overall, the decision to adjust fludrocortisone dose in this cohort of children with adrenal insufficiency was better supported by the biochemical parameters when based on renin concentration results and clinical status.

## Introduction

Patients with adrenal insufficiency are treated with glucocorticoid and mineralocorticoid replacement (1, 2). Plasma renin measured as either plasma renin activity (PRA) or plasma renin concentration (PRC) is used to evaluate whether the dose of fludrocortisone is appropriate (1, 3, 4, 5, 6). At our institution, a change in renin assay from activity to concentration measurement was implemented in 2015. This was done as obtaining reagents for the PRA assay had become unreliable, the assay curve flattened at high PRA values, making it difficult to assign a true value, and the PRC assay had become available for the iSYS analyser.

Following this change, concern was raised by the clinical team that a greater number of high renin concentrations (indicating a need to increase fludrocortisone dose) were being observed than had been the case for the activity assay. A service review was carried out to assess if appropriate biochemical control was achieved for patients receiving treatment with fludrocortisone, using sodium, potassium, renin and 17α-hydroxyprogesterone (17α-OHP) levels as markers (2).

## Materials and methods

The service review was carried out on patients with salt-wasting adrenal insufficiency, the majority having congenital adrenal hyperplasia (CAH). All were attending the regional adrenal clinic, run by the paediatric endocrine team with dosing decisions made by individual consultants based on clinical and biochemical parameters. Data on renin measurements were obtained retrospectively from the laboratory computer system (APEX). The assay methodology was changed from PRA to PRC in May 2015. Two cohorts were established: one comprised the last 50 PRA measurements made before the switch and the other comprised 50 PRC measurements made at the time the service review was initiated. A renin result was selected if there was an electrolyte profile taken at the same time. More than one sample from the same patient was included as long as it was on a separate occasion and was considered a separate fludrocortisone dose assessment event. The laboratory measures 15–20 renin samples per month from the Adrenal clinic, and this number remained the same with the assay change. The patient’s age, weight, height (to derive body mass index) and fludrocortisone dose at the time of sample and the dose once the renin level was available were recorded. Concurrent 17α-OHP concentrations were collected where available.

PRA was analysed using the Renin MAIA kit (Radim Diagnostics), a competitive radioimmunoassay. The lower limit of detection was 0.2 ng/mL/h, with a measurement range of 0.23–25 mg/mL/h, but with reduced reliability at levels >10 ng/mL/h. Coefficients of variation (CVs) were 12.3% at 1.9 ng/mL/h, 20.1% at 5.2 ng/mL/h and 16.2% at 19.3 ng/mL/h. PRC was analysed using the IDS-iSYS Direct Renin assay, a sandwich chemiluminescence immunoassay. The lower limit of detection was 1.8 mU/L, with linearity over the range 1.8–550 mU/L. CVs were 6% at 48 mU/L, 5% at 89 mU/L and 3.7% at 427 mU/L.

**Figure 1:**
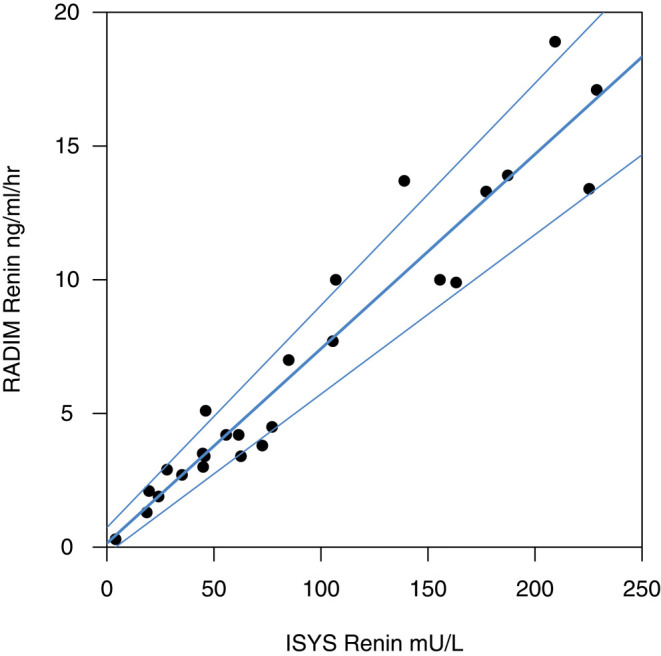
Comparison of the Radim Renin MAIA RIA, which measures renin activity, and the IDS-iSYS Direct Renin assay, which measures renin concentration from 27 patients with adrenal insufficiency. Passing–Bablok regression plot (with upper and lower confidence intervals) compares two different assay types using differences between them.

Previous comparison of the two renin assays in our institution showed they were significantly correlated (Spearman’s correlation = 0.92; *P* < 0.001) over ranges up to 20 ng/mL/h for PRA and up to 250 mU/L for PRC. As seen in a Passing–Bablok plot, the clustering of points close to the regression line is better for PRA values <10 ng/mL/h, consistent with higher PRA values being less reliable (Fig. 1). We used the regression equation from the validation study to convert PRA values to renin concentrations: renin concentration = 13.74 × (renin activity) – 2.52.

To correct for age, renin concentrations were converted to multiples of the upper limit of normal (MoU) using the formulae – age <1 year PRC/109.2, <2 years PRC/93.6, <10 years PRC/62.4 and <19 years PRC/31.2 mU/L.

Statistical analyses were carried out using SPSS (Ver25) with R (Ver 4.2.1) used for the Passing–Bablok plots. One-way ANOVA tests were used to compare between assay types, and Pearson’s chi-squared and Fisher’s exact tests for the effects of dose change by assay type and on renin MOU, electrolytes and 17α-OHP. The fludrocortisone dose was reduced in a minority of patients (9/100); therefore, in the chi-squared/Fisher’s tests, two groups were analysed – increased dose or no change to dose.

Spearman’s correlations were used to compare renin MOU with electrolytes, biochemical parameters and fludrocortisone dose.

In the PRA cohort, samples were taken from 40 patients who had CAH, four who had Addison’s disease, two who had adrenal insufficiency of unknown cause, two who had aldosterone synthase deficiency, one who had post-adrenalectomy adrenal insufficiency and one who had fludrocortisone dependency and postural hypotension.

In the PRC cohort, samples were taken from 34 patients who had CAH, ten who had Addison’s, three who had aldosterone synthase deficiency, two who had adrenal insufficiency of unknown cause and one who had triple A syndrome.

As this was a retrospective review/audit of results of routine clinical practice, neither patient consent nor ethical approval was required.

## Results

Biochemical data for the whole cohort and for each assay cohort are detailed in Table 1. Mean renin expressed as MoU was significantly different between the PRC (*n* = 50, mean renin MoU = 3.91 ± 4.67) and the PRA (*n* = 50, mean renin MoU = 2.05 ± 1.30; ANOVA *P* = 0.008) groups, whilst no significant differences were seen in any other parameters tested (Table 1).

**Table 1:** Demographic and biochemical data on the two cohorts by renin assay type (*n* = 50 in each).

	Renin concentration assay (mU/L)	Renin activity assay (ng/mL/h)
Age (years)	9.4 (5.7)	7.8 (6.1)
BMI (kg/m^2^)	19.2 (4.9)	19.5 (4.7)
Renin as MoU	**3.9 (4.7)**	**2.1 (1.3)^a^**
17α-OHP (nmol/L)	57 (93)	64 (42)
Fludrocortisone dose (µg)	155 (70)	146 (77)
Post-clinic fludrocortisone (µg)	172 (80)	154 (75)
Sodium (mmol/L)	138.3 (2.1)	139.1 (3.4)
Potassium (mmol/L)	4.1 (0.4)	4.2 (0.5)
Urea (mmol/L)	4 (1.1)	4.1 (1.4)
Creatinine (µmol/L)	42.3 (19.3)	40.2 (18.1)

Values are mean (s.d.) with significant differences between assays highlighted in bold (one-way ANOVA).

^a^ *P* = 0.008.

Significantly more children had their dose increased using the PRC (24/44, 55%) compared to the PRA (16/47, 34%, chi-squared *P* = 0.05 and Fisher’s test *P* = 0.04) (Table 2).

**Table 2:** Chi-squared analysis between assay type and fludrocortisone dose group. Comparison between observed counts in the fludrocortisone dose groups (increased, no change) and assay types showed significant associations between assay type and dose group by both Pearson’s chi-squared (*P* = 0.049) and Fisher’s exact test (*P* = 0.04).

Assay type	Counts	Dose group		Total
		Increased	No change	
Concentration assay	Observed	24	20	44
Activity assay	Observed	16	31	47
Total	Observed	40	51	91

**Figure 2:**
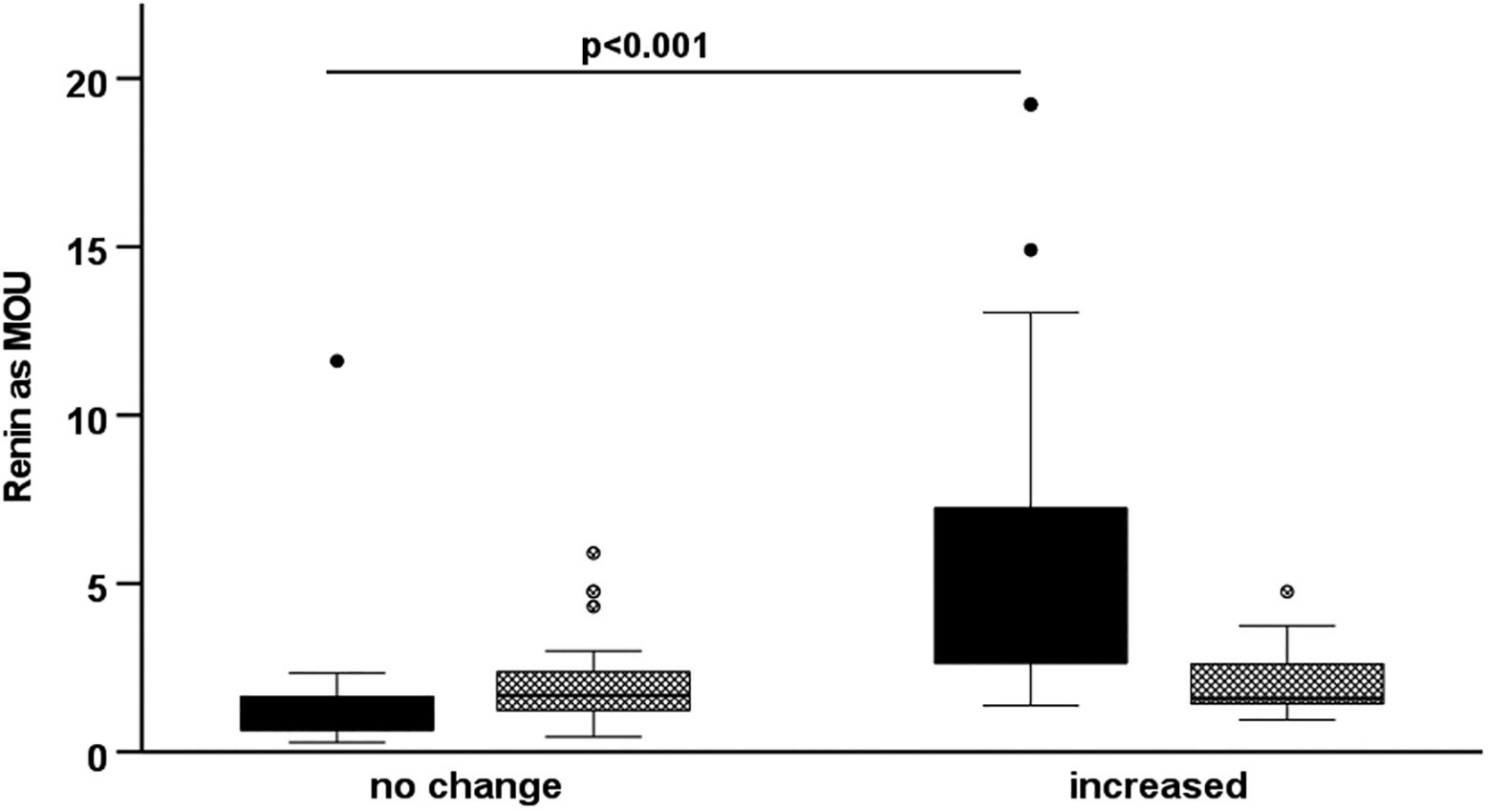
Renin levels expressed as MoU by change in fludrocortisone dose group and assay type. Concentration assays are represented by solid boxes and activity assays by hatched boxes. Boxes represent interquartile range (IQR) and whiskers represent 1.5 times the IQR. Outliers (between >1.5 and <3 times the IQR) are represented by filled or hatched circles. There was a significant difference in renin as a MoU by fludrocortisone dose group only for the renin concentration assay.

Comparison of the mean renin MoU between the increased fludrocortisone dose and the no-change groups showed a significantly higher level in the increased dose group for the PRC but not for the PRA (Fig. 2).

Renin MoU in the PRC group was positively correlated to fludrocortisone dose at the time of sampling (*r* = 0.38, *P* < 0.01) and to the post clinic dose, when adjustment may have been made (*r* = 0.64, *P* < 0.001) and negatively correlated to plasma sodium (*r* = −0.44, *P* < 0.001) (Table 3). However, the correlations for these parameters in the PRA group were weaker for the fludrocortisone doses, and not present for plasma sodium (Table 3).

**Table 3:** Correlations between renin MoU, electrolytes, 17α-OH-progesterone and fludrocortisone dose.

		Sodium	Potassium	17α-OHP	Fludrocortisone dose	Post-clinic fludrocortisone dose
PRC (MoU)	Correlation coefficient	−0.433	0.07	0.52	0.38	0.64
	*P*	**<0.001**	0.51	**<0.001**	**<0.01**	**<0.001**
PRA (MoU)	Correlation coefficient	−0.248	−0.06	0.56	0.29	0.32
	*P*	0.08	0.66	**<0.01**	**0.04**	**0.02**

Values represent Spearman’s correlation coefficients with significant correlations (*P* < 0.05) highlighted in bold.

In Table 4, the decision-making for fludrocortisone dosing related to renin as a MoU, blood 17α-OHP concentrations and plasma sodium for each assay was assessed. For the PRC assay, the fludrocortisone dose was more likely to be increased with MoU ≥1.5 and less likely to be changed for a 17α-OHP <40 mmol/L than for the PRA assay. For both assays, fludrocortisone dose was more likely to be increased for a plasma sodium <140 mmol/L, with this being more significant for the PRC.

**Table 4:** Chi-squared test between dose groups and renin MoU, 17α-OHP (nmol/L) and sodium (mmol/L) for renin concentration assay (A) and the renin activity assay (B).

		Renin MoU		17α-OHP (nmol/L)		Sodium (mmol/L)	
**Renin concentration assay**							
Fludrocortisone dose group		<1.5	≥1.5	< 40	≥40	<140	≥140
Increased dose	Count	2	22	6	7	24	0
No change	Count	14	6	12	1	11	9
	Chi-square	**<0.001**		**0.011**		**<0.001**	
	Fisher’s exact	**<0.001**		**0.015**		**<0.001**	
**Renin activity assay**							
Fludrocortisone dose group		<1.5	≥1.5	< 40	≥40	<140	≥140
Increased dose	Count	6	10	2	4	14	2
No change	Count	14	17	8	13	15	16
	Chi-square	0.62		0.83		**0.009**	
	Fisher’s exact	0.43		0.61		**0.009**	

Significant values for chi-squared and Fisher’s exact tests are highlighted in bold.

## Discussion

Plasma renin, measured as either an activity or as a direct concentration, is used to monitor the adequacy of fludrocortisone in controlling salt wasting. Decisions to change fludrocortisone dose are dependent on several factors – the degree of elevation of the renin value, the electrolyte levels (sodium and potassium), the clinical status of the child (2) and the level of 17α-OHP (in those with CAH). If the elevation is modest (between 1 and 1.5× the MoU) and other parameters are satisfactory, the clinical decision is likely to favour no change in dose.

However, a range of views are presented in the literature. High levels of PRA indicate a need to increase fludrocortisone dose, but low levels of PRA are not a reliable indicator for reducing doses (3). These authors recommended using low potassium as an indicator of over-replacement. We looked at potassium (results not shown) and found that it was not discriminatory in decision-making. In keeping with our clinical practice, Fiad *et al.* (4) found that elevated PRA in the context of other normal clinical and biochemical parameters is an indicator of adequate dose of mineralocorticoid. Finkielstain *et al.* (6) have previously recommended that titration of fludrocortisone aims to keep PRA in the middle of the reference range, and Husebye *et al.* (1) have recommended measuring PRA when there is suspicion of a mineralocorticoid-deficient state, though without a criterion for how to use it. However, it is thought that PRA lacks accuracy due to glucocorticoid-mediated changes in angiotensinogen levels and a resultant upward shift of PRA, hence a move towards using PRC (7). Whilst other studies have looked at using PRC (8, 9), there are no reports directly comparing the use of PRC and PRA for monitoring of patients with adrenal insufficiency.

Pofi *et al.* (5) have carried out a study to evaluate the dose of mineralocorticoid against various biochemical and clinical observations. This study found that in their childhood cohort, mineralocorticoid dose when corrected for body surface area (BSA) correlated to PRC, sodium and potassium. In their adult cohort, PRC correlated with total daily dose of fludrocortisone but not with the electrolytes. In contrast, in our dataset, correlations were found between PRC MoU and daily dose of fludrocortisone and with sodium and 17α-OHP but not potassium (Table 3). We recognise that, in our study, the correlation coefficients between PRC and fludrocortisone dose, sodium and 17α-OHP were modest but importantly higher than PRA, indicating that PRC is a more clinically relevant marker of biochemical status and fludrocortisone dose. When the fludrocortisone dose was adjusted for BSA, correlations to any of the parameter were not seen (data not shown). In our cohorts, dose correction by BSA compressed the range of fludrocortisone doses, inferring that the larger children were getting a similar dose per m^2^ compared with the smaller children. Notably, we found that the fludrocortisone dose after a decision to change the dose had been made was more strongly correlated to renin MoU (Table 3). Pofi *et al.* (5) also demonstrated in a multivariate model that renin is the dominant parameter and seems to overshadow all the other biochemical measurements.

In our study, when the change from PRA to PRC was made, it became apparent that renin values in comparable patient cohorts were higher with PRC, as shown by the difference in the median MoUs (Table 1). This resulted in 48% having fludrocortisone dose increases for PRC compared with 32% for PRA. This prompted a review to identify whether decisions to change fludrocortisone doses were being made appropriately. The analysis was undertaken by the laboratory team, who were blinded to the decision-making about dose changes. As the majority of the two cohorts had CAH, we used 17α-OHP and plasma sodium levels as comparators to the renin assays for decision-making. The focus was on the numbers whose fludrocortisone dose was increased or not changed. There was a minority who had their dose decreased – 12% for PRC and 6% for PRA. The larger numbers having either an increase or decrease in fludrocortisone dose for PRC indicate that this assay is more discriminatory for decision-making.

Assay results for PRA were converted to PRC and all results were then standardised to multiples of the upper limit of the PRC reference range to allow direct comparisons. This was justified based on the strong correlation between the two assays shown in Fig. 1.

The key finding that decisions to increase fludrocortisone were made more frequently with the PRC was supported by comparable analyses using sodium, whilst decisions not to change the dose based on 17α-OHP levels were also more frequent using the PRC. The clinical team was therefore satisfied that their decision-making for fludrocortisone dosing based on PRC had a stronger evidence base than when the PRA was being used. In common with previous work (8), and considering the various conditions included in this study, renin measurements are helpful for measuring the degree of mineralocorticoid insufficiency in all forms of adrenal insufficiency.

Our service review has several limitations. Individual clinicians made decisions to alter the dose of fludrocortisone, which would potentially introduce bias. However, although there was no defined protocol to make patient management decisions, the team have a consensus approach to altering fludrocortisone – this includes consideration of the clinical status of the child and whether there is a significant elevation in renin (>1.5 times the upper limit of normal) ± a lower sodium ± poor control as judged by 17α-OHP levels. We had to select two cohorts, one from the era of PRA and the other from the PRC era, so they are not the same patients. For both cohorts, they were selected in the same way as 50 contiguous assay results from the database. In addition, in both cohorts, most had a diagnosis of CAH and their age and growth data were similar. We also acknowledge the limitations in converting PRA measurements to PR concentration, but we were reassured by the significant correlation between the assays (Fig 1). Another limitation of this work is that the shift of method itself may have increased the focus on dose adjustments and therefore led to more dose increases. Ongoing team discussions about the use of PRC have not revealed any clinical management issues.

## Conclusion

In conclusion, the decisions to adjust fludrocortisone dose were better supported by the biochemical parameters (sodium and 17α-OHP) when based on plasma renin concentration results. This has provided reassurance to the clinical team that the PRC assay is appropriate for children with adrenal insufficiency. We suggest that using a parameter such as multiples of the upper limit of normal for PRC is valuable, and this should be tested in a prospective study.

## Declaration of interest

All authors declare there is no conflict of interest in this report.

## Funding

This work did not receive any specific grant from any funding agency in the public, commercial or not-for-profit sector.
